# Impact and Treatment of Sarcopenia in Patients Undergoing Radiotherapy: A Multidisciplinary, AMSTAR-2 Compliant Review of Systematic Reviews and Metanalyses

**DOI:** 10.3389/fonc.2022.887156

**Published:** 2022-05-26

**Authors:** Federica Medici, Alberto Bazzocchi, Milly Buwenge, Alice Zamagni, Gabriella Macchia, Francesco Deodato, Savino Cilla, Pierandrea De Iaco, Anna Myriam Perrone, Lidia Strigari, Stefania Rizzo, Alessio G. Morganti

**Affiliations:** ^1^ Department of Experimental, Diagnostic and Specialty Medicine-DIMES, Alma Mater Studiorum University of Bologna, Bologna, Italy; ^2^ Diagnostic and Interventional Radiology, Istituto di Ricovero e Cura a Carattere Scientifico (IRCCS) Istituto Ortopedico Rizzoli, Bologna, Italy; ^3^ Radiation Oncology Unit, Gemelli Molise Hospital-Università Cattolica del Sacro Cuore, Campobasso, Italy; ^4^ Medical Physics Unit, Gemelli Molise Hospital-Università Cattolica del Sacro Cuore, Campobasso, Italy; ^5^ Division of Oncologic Gynecology, Istituto di Ricovero e Cura a Carattere Scientifico (IRCCS) Azienda Ospedaliero-Universitaria di Bologna, Bologna, Italy; ^6^ Centro di Studio e Ricerca delle Neoplasie Ginecologiche (CSR), University of Bologna, Bologna, Italy; ^7^ Medical Physics Unit, Istituto di Ricovero e Cura a Carattere Scientifico (IRCCS) Azienda Ospedaliero-Universitaria di Bologna, Bologna, Italy; ^8^ Service of Radiology, Imaging Institute of Southern Switzerland, Ente Ospedaliero Cantonale (EOC), Lugano, Switzerland; ^9^ Radiation Oncology, Istituto di Ricovero e Cura a Carattere Scientifico (IRCCS) Azienda Ospedaliero-Universitaria di Bologna, Bologna, Italy

**Keywords:** literature review, radiotherapy, sarcopenia, prognostic factors, AMSTAR-2

## Abstract

**Background:**

Sarcopenia (SP) is defined as the quantitative and functional impairment of skeletal muscles. SP is commonly related to older age and is frequent in patients with cancer. To provide an overview of SP in patients treated with radiotherapy (RT) and to evaluate the current evidence, we analyzed the available systematic reviews and meta-analyses.

**Methods:**

Reviews were identified using PubMed, Scopus, and Cochrane library databases, without date restriction. Only systematic reviews and meta-analyses on the prognostic impact of SP and on any treatments aimed at reducing SP effect, in patients undergoing RT, were included in this review. The analyses not separately reporting the results in patients treated with RT were excluded. The quality assessment was performed using AMSTAR-2 (A MeaSurement Tool to Assess systematic Reviews).

**Results:**

From the 84 papers identified, five reviews met the inclusion criteria with four reports mainly including non-randomized trials. Three reviews on the effect of SP showed a significantly negative impact on overall survival in patients undergoing RT and/or chemoradiation for H&N cancers (HR: 1.63-2.07). Two reviews on interventional studies showed the possibility of 1) improving physical functions through nutritional and physical interventions and 2) avoiding muscle wasting by means of sufficient protein intake. The quality assessment of the included review showed that two and three analyses are classifiable as having low and moderate overall confidence rating, respectively.

**Conclusions:**

The analyzed reviews uniformly confirmed the negative impact of SP in patients with H&N tumors undergoing RT and the possibility of improving muscle mass and function through nutritional and physical interventions. These results justify further research on this topic based on a more uniform SP definition and on a complete evaluation of the potentially confounding parameters.

## Introduction

Sarcopenia (SP) is defined as the quantitative and functional impairment of skeletal muscles and is commonly related to older age (1). In addition, SP is also frequent in patients with cancer, particularly those with esophageal and lung tumors (1). In the mid-2000s, cancer-related SP was identified as a separate entity from cachexia in patients with cancer (2, 3). However, there is a significant correlation in several settings between SP and higher incidence of perioperative adverse events, chemotherapy-related toxicity, and worsened survival (1).

Three consensus statements on SP definition were published (4–6). To date, there is broad consensus on the methods to be used for assessing SP, but unique cutoffs values are still lacking. The latest review by Cruz-Jentoft and colleagues of the European Working Group on Sarcopenia (EWGOS) consensus statement reports several methods for identifying subjects with SP. These involve measurement of muscle strength, muscle mass, and physical performance (7). As for muscle mass, this is an indirect assessment of body muscle mass performed on the available imaging at different levels considered surrogates of total body distribution.

The interest in SP in patients undergoing radiotherapy (RT) began about 10 years ago when Dalal et al. reported 63% incidence of SP in RT-treated patients with locally advanced pancreatic tumors and a correlation between SP and outcome (8). Furthermore, subsequent studies showed significant correlations between SP and various outcomes, such as acute toxicity during chemoradiation in esophageal tumors (9) and survival in patients undergoing RT for cervical (10) and head and neck (H&N) (11) cancers.

At the same time, with the aim to improve RT personalization, interest has gradually grown in recent years in the development of predictive models based on several tumor-, patient-, and treatment-related parameters (12). Therefore, if the impact of SP in patients undergoing RT will be confirmed independently from other known prognostic factors, then the assessment of SP would be potentially useful to develop new and more efficient predictive models.

In recent years, several studies have been published on the impact of SP in the RT setting. In addition, some systematic literature reviews and meta-analyses on this topic have been published over the past two years (13–17). However, no randomized studies have been published on this topic. Furthermore, no guidelines on the evaluation and management of SP in RT are currently available. Therefore, to provide an overview on SP in RT patients and to evaluate the available evidence, we analyzed the systematic reviews and meta-analyses currently published based on the AMSTAR-2 (A MeaSurement Tool to Assess systematic Reviews) guidelines (18).

## Materials and Methods

This literature review is part of the AFRAID (impAct oF saRcopeniA In raDiotherapy) project, and it was performed by a multidisciplinary team including radiation oncologists (FM, AZ, GM, FD, and AGM), radiologists (AB and SR), medical physicists (SC and LS), and cancer surgeons (AMP and PDI). The review process was based on the guidelines provided by Smith et al. (19).

### Eligibility Criteria

Only systematic reviews and meta-analyses on the prognostic impact of SP and on any treatments aimed at reducing its effect in patients undergoing RT were included in this review. Therefore, the analyses not separately reporting the results in patients undergoing RT were excluded. Moreover, papers written in a language other than English and conference abstracts were excluded. The selection of papers was performed regardless of the RT purpose (curative and palliative treatment of oligometastatic patients).

### Bibliographic Search

A literature search was performed without time limits, on December 15 2021, using the following bibliographic databases: PubMed, Scopus, and Cochrane library. Details of the search strategies in the different databases are given in **Supplementary Materials 1**. After the bibliographic research in the different databases, the duplicates were removed. Thereafter, the remaining sources were independently evaluated at title and abstract level by two different authors (MB and AZ), with the subsequent elimination of papers considered as not relevant. The remaining studies were evaluated, by the same two authors, by reading the entire text and excluding the papers not fitting the inclusion criteria.

### Data Extraction

The remaining papers were independently examined by three authors (GM, SC, and FD) to extract the following data: authors, year of publication, type of study (systematic review and/or meta-analysis), number and type of studies included in the analysis, main endpoints, analyzed parameters (on SP and body composition), and main and secondary results of the study. Any differences were resolved by consulting the senior author (AGM) during both paper selection and data extraction.

### Quality Assessment

The quality of the analyses included in this review was independently performed, using the AMSTAR-2 tool (14) by two different authors (FM and AZ). The overall confidence rating, based on AMSTAR-2 guidelines, was defined as follows: i) “high”, in case of 0–1 non-critical weakness; ii) “moderate”, in case of > 1 non-critical weaknesses; iii) “low”, in case of 1 critical flaw ± non-critical weaknesses; and iv) “critically low”, in case of > 1 critical flaw ± non-critical weaknesses.

### Data Analysis

Considering the large heterogeneity of the studies included in this review, in terms of selection criteria, endpoints, and analyzed parameters, we did not perform a quantitative analysis (meta-analysis). Instead, we limited ourselves to report in a single document the studies’ findings, consistency, and quality to provide a summary useful for clinical practice and to guide future research.

## Results

**Figure 1:**
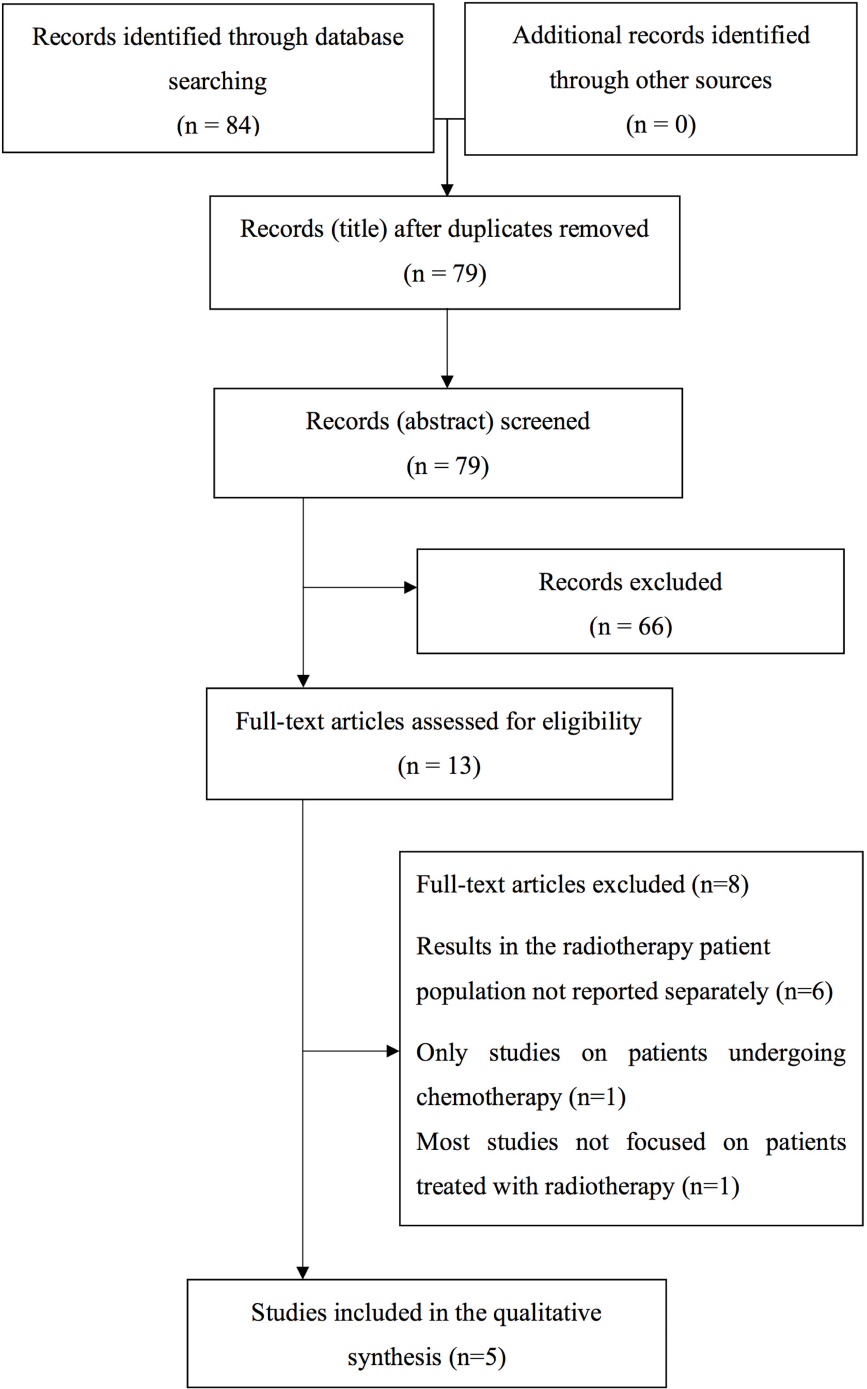
Process of paper selection.

Overall, the literature search provided 84 papers. After removing the duplicates, the remaining bibliographic sources were evaluated at title and abstract level with subsequent exclusion of 66 papers considered as not relevant. The remaining 13 analyses were evaluated by reading the entire text and further eight papers were removed. A list of the papers excluded from the revision (with the reasons of the ineligibility) after evaluation of the entire text is reported in **Supplementary Materials 2**. Consequently, five papers were selected for the aim of this review (**Figure 1**).

All retrieved studies were published in the past two years. Apart from the review by Bye et al., the other studies mainly included non-randomized trials (13, 15–17). Four reviews included only studies on patients with H&N tumors (13–16), whereas one study included reports on other tumor settings (17). Three reviews regarded the prognostic impact of SP in patients treated with RT (13, 15, 16), whereas two reviews reported the results of studies on possible interventions aimed at counteracting the effects of SP in the same setting (14, 17). The evaluation of SP in the reports included in the analyzed reviews was mainly based on CT (13, 15, 16). Furthermore, the two reviews on interventional studies evaluated other parameters regarding nutritional status and physical fitness (14) or muscle mass (17). Three reviews showed a significantly negative impact of SP on overall survival in patients undergoing RT and/or chemoradiation for H&N cancers (13, 15, 16). Regarding overall survival, the reviews by Takenaka et al. (16) and Findlay et al. (13) reported HR: 1.63 (95% CI: 1.40–1.90) and HR: 2.07 (95% CI: 1.47–2.92) in patients with SP compared to others, respectively. Furthermore, the two reviews on interventional studies showed the possibility of improving physical functions through nutritional and physical interventions (14) and avoiding muscle wasting by means of sufficient protein intake (17). The characteristics and results of the analyzed reviews are summarized in **Table 1**.

**Table 1:** Characteristics and results of the included studies.

Authors,year	Study type	Included studies, number/type	Main endpoints of the review	Analyzed parameters	Main findings	Other findings and notes
Bye A et al., 2020 (14)	SR and MA	13 RCTs: nine full-scale RCTs and four pilot RCTs	Effect of nutritional and physical interventions (alone and combined) during RT for patients with H&NC	Nutritional status: BMI, change in body weight, muscle mass, lean body mass or fat mass Physical exercise: fitness tests like walk test, handgrip strength, or changes in physical performance	Nutrition and physical exercise interventions have a positive effect on physical function while no effect was recorded in terms of nutritional status	Studies on combined interventions (nutritional and physical; all pilot RCTs) showed no effect compared to control groups
Findlay M et al., 2020 (15)	SR	11 observational cohorts: 10 retrospective and one prospective	Prognostic impact of SP in patients undergoing curative RT ± other treatments for H&NC	CT from PET-CT scans (six studies), abdominal or head and neck CT (four studies), and MRI (one study). Level of evaluation: L3 (seven studies), C3 (three studies), and T2 (one study)	Pre- and post-treatment SP and change in skeletal muscle status are associated with worse OS	The authors scored the certainty of evidence as “low”, for overall survival, locoregional control, and progression/disease-free survival and “very-low” for distant metastasis, RT interruptions, and chemotherapy-related toxicity, according to the Grading of Recommendations, Assessments, Development and Evaluation (GRADE) criteria
Takenaka Y et al., 2021 (16)	MA	11 retrospective cohort studies	Prognostic impact of SP in patients with H&NC treated with surgery or RT	SMI-L3 (eight studies) and SMI-C3 (ten studies)	SP is associated with worse disease-free survival, disease-specific survival, and OS, regardless of treatment modality	The impact of SP is stronger in patients undergoing surgery compared to RT. Not significant differences between sites of SMI definition (C3 vs. L3)
Capitão C et al., 2021 (17)	SR	One RCT, one “uncontrolled experimental”, and six observational	Optimal protein intake to maintain the muscle mass in patients with cancer during RT/CRT (H&NC: five studies; lung cancer: two studies, esophageal cancer: one study)	Indirect assessment of muscle mass: MAMC, BIA, FFMI, and CT	A protein intake > 1.2–1.4 g/kg per day (higher than the recommended 1.0 and 1.2 g/kg range) is needed to avoid muscle wasting during RT or CRT	Only one study used CT for muscle mass evaluation
Findlay M et al., 2021 (13)	SR and MA	Seven observational: six retrospective and one prospective	Prognostic impact of CT-defined SP on OS in patients with H&NC undergoing curative RT ± other treatment modalities	PET-CT or CT evaluated at L3 level	Pre- and post-treatment SP is associated with worse OS	The authors scored as “low” and “moderate” the certainty of evidence, for overall survival, regarding pre- and post-treatment SP, respectively, according to the Grading of Recommendations, Assessments, Development, and Evaluation (GRADE) criteria

BIA, bioelectrical impedance; BMI, body mass index; C3, third cervical vertebra; CRT, concurrent chemoradiation; CT, computed tomography; FFMI, fat-free mass index; H&NC, head and neck cancer; L3, third lumbar vertebra; MA, metanalysis; MAMC, mid-arm muscle circumference; OS, overall survival; PET, positron emission tomography; RCT, randomized-controlled trial; RT, radiotherapy; SMI, skeletal muscle index; SP, sarcopenia; SR, systematic review; T2, second thoracic vertebra.

On the basis of the assessment of the quality of the studies shown in **Supplementary Table 1**, two (14, 17) and three (13, 15, 16) reviews were classified as low and moderate overall confidence rate, respectively. The AMSTAR-2 domain with the highest number of critical weaknesses was “duplicated data extraction”.

## Discussion

Between 20% and 70% of patients with cancer suffer from SP (20). Moreover, SP resulted as an independent predictor of postoperative complications, chemotherapy-induced toxicity, and poor OS in a systematic review of 35 cancer studies (1). Recent findings highlighted the benefits of early identification and management of SP in patients with cancer. However, the definition of optimal nutritional and pharmacological approaches to SP is still in a preliminary stage (21).

Our study focused on SP in RT-treated patients, and from the analyzed systematic reviews and meta-analyses, we found uniform evidence about the significant impact of SP on prognosis. This finding is important and suggests the inclusion of SP assessment in future personalized RT strategies. Furthermore, this result stimulates the evaluation of SP in patients undergoing RT to generate further data and analyses useful to draft clinical guidelines and to design new predictive models.

Among the assessment methods reported in the reviewed systematic reviews and meta-analyses (13, 15–17), the skeletal muscle mass method predominates, except for Bye and colleagues (14) where muscle strength and physical performance were also used.

The magnitude of the impact of SP on overall survival is particularly noteworthy. In fact, regarding overall survival, Takenaka et al. (16) and Findlay et al. (13) reported HR: 1.63 (95% CI: 1.40–1.90) and HR: 2.07 (95% CI: 1.47–2.92) in patients with SP compared to others, respectively. It is interesting to compare these figures with the quantitatively lower benefits achievable in H&N tumors from the combination of RT with chemotherapy (HR: 0.90) (22) or with cetuximab (HR: 0.74) (23).

This literature review has several limitations. First, apart from the meta-analysis by Bye et al., all the other reviews included mainly non-randomized studies. Moreover, the many concerns about the conduct and reporting of systematic reviews of non-randomized studies are well known (18, 24). Furthermore, our review clearly shows the strong heterogeneity in SP definition. For example, Findlay et al. (15) reported eight different SP definitions out of the eleven analyzed studies. Moreover, the prognostic impact of possible confounding factors, together with that of SP, was poorly evaluated. For example, in the second analysis by the same authors (13), only one out of seven studies considered the performance status among the adjustment factors and three analyses (13, 14, 17) did not include the impact of tumor stage. On the contrary, a combined evaluation of SP and these parameters is obviously needed to clearly establish the independent impact of SP. In fact, the close correlation between performance status and SP in lung tumors (25) and between clinical tumor stage and SP in several cancers is well known (26). Another example comes from the review by Takenaka et al., where only one out of 11 study evaluated HPV status as a covariate (16). However, it is known that positive HPV patients generally show better clinical conditions (and therefore nutritional status) than negative HPV subjects and, at the same time, a better prognosis especially after RT. Therefore, it can be hypothesized that these issues could have influenced the impact of SP on overall survival. Furthermore, the quality assessment of the studies included in this review showed that two (14, 17) and three (13, 15, 16) out of five analyses are classifiable as having low and moderate overall confidence rating, respectively. Finally, the evidence included in our review mainly concerned H&N cancers, whereas no systematic reviews are currently available on the impact of SP in other settings.

Only in the study by Capitão et al. a report on esophageal cancers was included, demonstrating a high prevalence of malnutrition among patients with esophageal cancer, which worsened during concurrent chemoradiation (27) and two analyses on lung tumors reporting the association between oral protein intake and increased likelihood of maintaining the skeletal muscle mass (28).

However, beyond these limitations, three studies uniformly confirmed the negative impact of SP in patients with H&N tumors undergoing RT (13, 15, 16), and two studies uniformly confirmed the possibility of improving muscle mass and function through nutritional and physical interventions (14, 17). Therefore, if these preliminary results will be confirmed by more robust evidence, then the evaluation of SP before RT, especially in patients with H&N tumors, will allow to: i) implement SP treatment and prevention strategies during RT; ii) design trials on RT specifically adapted to the risk of tumor relapse (based on SP presence and grade together with other prognostic factors) with RT dose-de-escalation protocols in low-risk patients and RT dose-escalation in patients at high risk; and iii) select patients to be treated with RT rather than other treatments; in this regard, for example, the review by Takenaka et al. showed a greater SP impact on overall survival in patients undergoing surgery than in subjects undergoing RT (16). If this data will be confirmed by other analyses, then it could lead to preferentially choosing RT, rather than surgery, in patients with sarcopenia.

Moreover, in our opinion, these results justify further research on this topic, hopefully based on a more uniform SP definition and on a complete evaluation of the potentially confounding parameters. These studies could be aimed at: i) analyzing the impact of SP in other tumor settings; ii) include SP into predictive models based on old and new prognostic factors, such as inflammation indices (29); iii) analyze the impact of SP together with standard nutritional indices (30); iv) specifically analyze the impact of sarcopenic obesity, a variant of SP including low muscle mass and high fat mass (31) and independently associated with worse survival and complication rates from local and systemic therapies, sometimes more significantly compared to SP alone (32); and v) evaluate the impact of SP not only on OS but also on the pattern of failures, to allow treatment modulation based on the risk of local relapses and distant metastases. Indeed, among the reviews included in our analysis, only Findlay et al. (15) reported data on locoregional control in patients with SP undergoing RT for H&N cancer, showing conflicting results between the analyzed papers.

These future studies will probably be simplified and accelerated by the use of Artificial Intelligence techniques for the detection of patients with sarcopenia (33).

## Author Contributions

FM, SR, LS, and AM had the idea for the article; MB, AZ, GM, SC, and FD performed the literature search and data collection and analysis; FM, AB, MB, PDI, AMP, and AGM drafted the manuscript. All authors contributed to the article and approved the submitted version.

## Conflict of Interest

The authors declare that the research was conducted in the absence of any commercial or financial relationships that could be construed as a potential conflict of interest.

## Publisher’s Note

All claims expressed in this article are solely those of the authors and do not necessarily represent those of their affiliated organizations, or those of the publisher, the editors and the reviewers. Any product that may be evaluated in this article, or claim that may be made by its manufacturer, is not guaranteed or endorsed by the publisher.
