# Supplementary material for: Impact and Treatment of Sarcopenia in Patients Undergoing Radiotherapy: A Multidisciplinary, AMSTAR-2 Compliant Review of Systematic Reviews and Metanalyses
Source: Front Oncol. 2022 May 26;12:887156. doi: 10.3389/fonc.2022.887156 (PMC9177942; doi:10.3389/fonc.2022.887156)
Supplement: Supplementary file 1 [file DataSheet_1.docx]

**SUPPLEMENTARY MATERIAL.**

**1. SEARCH STRATEGIES**

**PubMed:**

("sarcopenia" OR "body composition") AND ("radiotherapy" OR "radiation" OR "chemoradiation" OR "radiochemotherapy" OR "SBRT" OR "stereotactic radiotherapy" OR "SABR"); ONLY: “systematic review” OR “metanalysis”

**Scopus**

TITLE-ABS-KEY ( ( "sarcopenia" OR "body composition" ) AND ( "radiotherapy" OR "radiation" OR "chemoradiation" OR "radiochemotherapy" OR "SBRT" OR "stereotactic radiotherapy" OR "SABR" ) AND ( "systematic review" OR "metanalysis" ) ) AND NOT "DBCOLL(medl)"

**Cochrane library**

("sarcopenia" OR "body composition") AND ("radiotherapy" OR "radiation" OR "chemoradiation" OR "radiochemotherapy" OR "SBRT" OR "stereotactic radiotherapy" OR "SABR") AND (“systematic review” OR “metanalysis”) in Title Abstract Keyword - (Word variations have been searched)

**2. EXCLUDED STUDIES AFTER FULL TEXT ASSESSMENT (with reasons)**

- Wang Y, Tian G, Chen S, Li N. Myosteatosis reduces overall survival in patients with digestive system malignancies: a meta-analysis with trial sequential analysis. Nutr Res. (2021); 94:25-33. doi: 10.1016/j.nutres.2021.08.003. Epub 2021 Aug 23. PMID: 34583210. (results in the radiotherapy patient population not reported separately)
- Lopez P, Newton RU, Taaffe DR, Singh F, Buffart LM, Spry N, Tang C, Saad F, Galvão DA. Associations of fat and muscle mass with overall survival in men with prostate cancer: a systematic review with meta-analysis. Prostate Cancer Prostatic Dis. (2021). doi: 10.1038/s41391-021-00442-0. Epub ahead of print. PMID: 34420038. (results in the radiotherapy patient population not reported separately)
- Furbetta N, Comandatore A, Gianardi D, Palmeri M, Di Franco G, Guadagni S, Caprili G, Bianchini M, Fatucchi LM, Picchi M, Bastiani L, Biancofiore G, Di Candio G, Morelli L. Perioperative Nutritional Aspects in Total Pancreatectomy: A Comprehensive Review of the Literature. Nutrients. (2021);13:1765. doi: 10.3390/nu13061765. PMID: 34067286; PMCID: PMC8224756. (results in the radiotherapy patient population not reported separately)
- Klement RJ, Brehm N, Sweeney RA. Ketogenic diets in medical oncology: a systematic review with focus on clinical outcomes. Med Oncol. (2020);37:14. doi: 10.1007/s12032-020-1337-2. PMID: 31927631. (the review included only studies on patients undergoing chemotherapy)
- de Aguiar Pastore Silva J, Emilia de Souza Fabre M, Waitzberg DL. Omega-3 supplements for patients in chemotherapy and/or radiotherapy: A systematic review. Clin Nutr. (2015);34:359-66. doi: 10.1016/j.clnu.2014.11.005. Epub 2014 Nov 14. PMID: 25907586. (most studies were not focused on patients treated with radiotherapy)
- Hua X, Liu S, Liao JF, Wen W, Long ZQ, Lu ZJ, Guo L, Lin HX. When the Loss Costs Too Much: A Systematic Review and Meta-Analysis of Sarcopenia in Head and Neck Cancer. Front Oncol. (2020);9:1561. doi: 10.3389/fonc.2019.01561. PMID: 32117787; PMCID: PMC7012991. (results in the radiotherapy patient population not reported separately)
- Jahrreiss V, Laukhtina E, D'Andrea D, Shariat SF. The prognostic value of sarcopenia in patients with prostate cancer: a systematic review. Curr Opin Urol. (2021);31:315-323. doi: 10.1097/MOU.0000000000000885. PMID: 33965982. (results in the radiotherapy patient population not reported separately)
- Ferrão B, Neves PM, Santos T, Capelas ML, Mäkitie A, Ravasco P. Body composition changes in patients with head and neck cancer under active treatment: a scoping review. Support Care Cancer. (2020);28(10):4613-4625. doi: 10.1007/s00520-020-05487-w. Epub 2020 Jun 13. PMID: 32533436. (results in the radiotherapy patient population not reported separately)

**TABLE S1:**Summary of quality evaluation of the included reviews based on AMSTAR 2 domains.

| **Authors, publication year** | **Bye**  **2020** | **Findlay 2020** | **Takenaka**  **2021** | **Capitao 2021** | **Findlay 2021** |
| --- | --- | --- | --- | --- | --- |
| 1. Review question (PICO)[*^a^*](https://onlinelibrary.wiley.com/doi/10.1111/jebm.12423#jebm12423-tbl1-note-0001_29) | Y | Y | Y | Y | Y |
| 2. A priori protocol | Y | Y | Y | Y | Y |
| 3. Study designs included/explained | N | N | N | N | N |
| 4. Comprehensive literature search[*^a^*](https://onlinelibrary.wiley.com/doi/10.1111/jebm.12423#jebm12423-tbl1-note-0001_30) | P | Y | Y | Y | P |
| 5. Duplicate study selection[*^a^*](https://onlinelibrary.wiley.com/doi/10.1111/jebm.12423#jebm12423-tbl1-note-0001_31) | Y | Y | Y | Y | Y |
| 6. Duplicate data extraction[*^a^*](https://onlinelibrary.wiley.com/doi/10.1111/jebm.12423#jebm12423-tbl1-note-0001_32) | N | Y | Y | N | Y |
| 7. List and justification of excluded studies | N | N | N | N | N |
| 8. Adequate description of included studies | Y | Y | Y | Y | Y |
| 9. Satisfactory technique for assessing RoB in nonrandomized studies[*^a^*](https://onlinelibrary.wiley.com/doi/10.1111/jebm.12423#jebm12423-tbl1-note-0001_33) | Y | Y | Y | Y | P |
| 10. Funding sources reported for included studies | N | N | N | N | N |
| 11. Appropriate methods for quantitative synthesis | Y | NMA | Y | NMA | Y |
| 12. Impact of RoB on pooled results[*^a^*](https://onlinelibrary.wiley.com/doi/10.1111/jebm.12423#jebm12423-tbl1-note-0001_34) | Y | NMA | Y | NMA | Y |
| 13. Impact of RoB on interpretation of pooled results[*^a^*](https://onlinelibrary.wiley.com/doi/10.1111/jebm.12423#jebm12423-tbl1-note-0001_35) | Y | Y | Y | Y | Y |
| 14. Heterogeneity explained[*^a^*](https://onlinelibrary.wiley.com/doi/10.1111/jebm.12423#jebm12423-tbl1-note-0001_36) | Y | Y | Y | Y | Y |
| 15. Publication (small study) bias assessed[*^a^*](https://onlinelibrary.wiley.com/doi/10.1111/jebm.12423#jebm12423-tbl1-note-0001_37) | Y | NMA | Y | NMA | Y |
| 16. Funding sources and conflict of review authors reported | Y | Y | Y | Y | Y |

*Legend*: a Key domains for overall confidence ratings of adherence-outcomes reviews; N, no; NMA: No meta-analysis conducted; P, partial yes; PICO, population, indication, comparator, outcomes; RoB, risk of bias.; Y, yes.

- Range and meaning of overall confidence ratings based on AMSTAR 2.
- “High - Zero or one non-critical weakness: The systematic review provides an accurate and comprehensive summary of the results of the available studies that address the question of interest.
- Moderate - More than one non-critical weakness*: The systematic review has more than one weakness, but no critical flaws. It may provide an accurate summary of the results of the available studies that were included in the review.
- Low - One critical flaw with or without non-critical weaknesses: The review has a critical flaw and may not provide an accurate and comprehensive summary of the available studies that address the question of interest.
- Critically low - More than one critical flaw with or without non-critical weaknesses: The review has more than one critical flaw and should not be relied on to provide an accurate and comprehensive summary of the available studies
